# Do weak readers in rural India automatically read same language subtitles on Bollywood films? An eye gaze analysis

**DOI:** 10.16910/jemr.15.5.4

**Published:** 2022-11-24

**Authors:** Somnath Arjun, Brij Kothari, Nirav Kumar Shah, Pradipta Biswas

**Affiliations:** Indian Institute of Science, Bangalore, India; Indian Institute of Management, Ahmedabad, India; PlanetRead, Pondicherry, India

**Keywords:** Eye-tracking, Same Language Subtitling, Eye Movements, Reading, Literacy, Attention

## Abstract

Same Language Subtitling (SLS) of audio-visual content on mainstream TV entertainment to improve mass reading literacy was first conceived and piloted in India. SLS is now being scaled up nationally to ensure that the reading skills of one billion TV viewers, including 600 million weak readers, remain on a lifelong pathway to practice, progress, and proficiency. Will weak readers ignore or try to read along with SLS? Our eye-tracking study investigates this question with 136 weak readers drawn from a remote village in Rajasthan state by showing them popular Hindi film clips of dialog and songs, with and without SLS. We developed an interactive web-based visual analytics tool for exploring eye-tracking data. Based on an analysis of fixations, saccades, and time spent in the subtitle and non-subtitle areas, our main finding is that 70 percent of weak readers engaged in unprompted reading while watching film clips with SLS. We observed that saccadic eye movement is a good indicator to quantify the amount of reading with SLS, and saccadic regression can further differentiate weak readers. Eye-tracking studies of weak readers watching subtitles are rare, and ours may be the first with subjects from rural India.

## Introduction

Ninety-five percent of the world’s illiterate people live in developing countries (54). India’s literacy rate is 78 percent (40) but the quality of literacy is extremely low. Sixty percent “literates” cannot read simple texts, much less a newspaper (26). Children fall behind in reading from the early grades and weak skills only erode later in life due to a lack of reading practice. The nation and practically every state confronts weak reading at scale and the overwhelming majority of non-literates and weak readers are rural and female (10). The long-term goal of the Billion Readers (BIRD) initiative at the Indian Institute of Management, Ahmedabad (IIM-A) is to ensure daily and lifelong reading practice for a billion people in India. In order to achieve this goal, BIRD aims to scale up Same Language Subtitling (SLS) on all existing entertainment content on television and streaming platforms, expressly for reading literacy practice and skill improvement at population scale. This is a global first in some important respects.

Till date, no country’s TV and/or streaming policy has implemented text on screen explicitly for mass reading literacy. Several majority English-speaking countries have implemented captions for media access among the Deaf and Hard or Hearing (DHH) (38). Since Price, (45 first researched the availability of captions for learning English as a Second Language (ESL), a large body of evidence has supported captions for second language learning (Burger, 8 maintains a comprehensive online bibliography).

The suggestion of leveraging captions for reading literacy among the DHH (9) and the hearing (23) is as old as the idea itself. However, the use of captions to strengthen reading has been far less researched than their use for language learning and has certainly not driven national broadcast policy on mainstream TV or streaming, anywhere in the world, for mass reading literacy. The ‘SLS’ project was first conceived in India in 1996 with the intent to move national media policy and impact reading literacy at scale (25). Existing terms like ‘bimodal’ and ‘intralingual’ subtitling, while meaning exactly the same, did not suit the project’s need for a self-evident term that spoke widely to policy makers and viewers alike.

SLS is the idea of subtitling audio-visual (AV) content in the ‘same’ language as the audio. What you hear, is what you read. SLS means Hindi subtitles on Hindi content, Tamil subtitles on Tamil content and likewise on all existing and popularly watched Indian language content like films, serials, cartoons, and songs. In India and globally, most entertainment content in English is available with SLS. That is not the case on content in most of the world's other, and especially, non-Roman script languages. Streaming platforms in India offer translation subtitles in a number of languages, but not in the ‘same’ audio language.

SLS is a deceptively simple social innovation with the power to transform a large population of struggling readers into functional and even fluent readers. Globally, the enormous value of SLS for reading literacy at population scale remains mostly untapped. After field testing in villages and poor urban communities, researchers at IIM-A piloted SLS on TV in Gujarat state in 1999 and found it to be an effective intervention for improving reading skills (29). Since then, other studies have confirmed that frequent matching text-sound exposure improves reading (e.g., 27).

The theory of change driving BIRD is that a population that engages in reading every day, all through life, cannot remain weak reading. Currently there is nothing that ensures daily reading practice for all Indians, across the average lifespan of 70 years. If all the entertainment content on TV and streaming platforms carried SLS, millions of viewers would automatically try and associate the matching text and sound. Reading skills would remain in a constant state of reinforcement.

To our knowledge eye-tracking studies of beginning or weak-readers’ viewing of subtitles are rare, if any, anywhere in the world. Most eye-tracking studies on dynamic texts assume functionally reading subjects and hardly any have been conducted in a developing country (32). Negi & Mitra (39) study in India is with good readers with a minimum 10^th^ Grade education. Our present eye-tracking study in rural Rajasthan, with weak readers – those who can decode some letters or simple words but struggle to read the words in a 2^nd^ Grade level text as single units, i.e., – they lack the ability to unitize (14) – may be a first.

An eye-tracking study undertaken earlier (31) found that viewers who saw videos with SLS and read them had better comprehension than those who saw the same videos but did not read the SLS. The participants were literate college students in South Africa who spoke English as a second language. The study was conducted in the context of English subtitles on academic lectures delivered in English. It demonstrated the educational potential of SLS in reading instruction and language learning. In rural India, the primary source of entertainment is via native language films and television programs. Could SLS on entertainment content be an approach to improved reading skills?

There is some evidence in the literature that SLS significantly impacts reading skills (5; 11; 36). A controlled experiment with schoolchildren in India demonstrated that SLS leads to reading improvement (30). A national TV pilot of reading improvement further supported that there is a significant effect of SLS on reading skills (27).

Most of the analyses for previous studies focused on the change in reading performance but did not eye-track the reading engagement with SLS. This paper analyzes the processing of SLS on popular Hindi songs and dialogs with eye gaze metrics. In addition to fixation and scan path measures, we investigated regressions, saccade amplitudes, and revisits. These metrics helped us to examine the reading behavior of participants and the proportion of engagement with SLS. We developed a visual analytic tool to investigate eye-tracking data. The tool provided information about eye gaze patterns that would have gone unnoticed otherwise. For example, the tool could differentiate between the reading pattern of weak readers who can read with difficulty (functional weak readers) and very weak readers who cannot read functionally (poor readers) with respect to subtitle reading in videos with SLS.

We discuss the related work on SLS in section 2 followed by an overall design of our user study in section 3. The methodology is discussed in section 4. Section 5 presents our analysis and results followed by concluding remarks in section 6.

## Related Work

The most sustained push for SLS on TV for reading literacy has been in India, through research, pilot TV implementations in 10 Indian languages and evidence-based policy advocacy (28). However, the positive impact of SLS use on reading literacy has also been affirmed in research studies from other countries (24; 35, 36; 41). Taken together, these studies make a strong case that routine and regular exposure to SLS can have an impact at scale on a population’s reading development.

Previous studies have found that the presence of SLS on popular entertainment content causes automatic reading skill practice and improvement among viewers who can minimally recognize letters (29; 27). Moreover, there is compelling evidence that SLS also promotes language learning and media access among the Deaf and Hard of Hearing (DHH) (11; 17; 43; 53). While any of these benefits provides a strong rationale for SLS, the evidence for all three makes it a compelling proposition on TV and streaming platforms.

SLS on popular entertainment content is a critical aspect of BIRD for two factors that powerfully drive reading skill acquisition: first, an inordinate amount of practice and second, with text embedded in a context that the reader will be passionate about for life. Toste et al. (50) review the powerful bi-directional relations between motivation and reading skill. Both drive each other. For proficient reading, grapheme-phoneme associations need to fire frequently and sufficiently over a long enough period of time to achieve and sustain automaticity. As Frey & Fisher (16) state “When we experience something, neurons fire. Repeated firings lead to physical changes in the brain that, over time and with repetition, become more permanent.” They further point out that “The challenge, of course, with automaticity is to not allow repetition to turn into a rut.”

When reading acquisition occurs in a person’s first language, the auditory-to-language pathways are well-established in the brain. In a weak reader, the letter-to-sound-to-meaning pathways are weak (52). Reading fluency and comprehension are achieved over a long period of sustained exposure to congruent letter-sound correspondence and decoding practice. As decoding approaches fluency and automaticity, cognitive resources are freed to focus on the key task of meaning-making. SLS on popular songs, nursery rhymes and repeatedly watched cartoons has the added advantage of reading practice with predictable sound-to-text reinforcement, on content of high-interest (19; 22). The visual and auditory pathways involved in reading are strengthened incidentally, subconsciously, and inevitably, as a by-product of entertainment.

There are few longitudinal studies on the impact of captions on reading skills (23). Linebarger et al. (36) study indicated that children who viewed video with captions improved their reading faster than their counterparts who viewed without captions, and the improvement was most pronounced among children at risk for poor reading outcomes. Similarly, in New Zealand, Parkhill & Johnson (42) found that in their six-week ‘AVAILLL’ program for children aged 5-13 years, which uses popular, subtitled movies and accompanying novels to engage students in reading literacy, the greatest gains occurred for ‘low-progress’ readers. A positive impact was also observed for average and higher-level readers. Kothari & Bandyopadhyay (27) evaluated the impact of SLS after sustaining it for 5 years on a weekly hour-long program of Hindi film songs telecast nationally in prime time. Among school children who could not read a single letter in Hindi at the baseline (2002), 70% in the high-SLS viewing group became functional readers by the end line (2007) as compared to 34% in the low-SLS group. The benefits of SLS or Closed-Captioning are not limited to reading literacy. The range of benefits attributable to SLS include reading, media access and language acquisition (17).

Although there is a substantial body of research on measuring the effect of SLS on reading using standardized tests, eye-tracking studies that focus on the development of reading skills are uncommon (46). Studies have examined gaze patterns while reading text or phrases (4; 18; 37). Smyrnakis et al. (49) compared the silent and loud reading ability of typical and dyslexic readers using eye-tracking while Faber et al. (15) investigated the differences in stable eye movement patterns during narrative reading. Eye-tracking studies were also used to understand cognitive load while reading (2; 33).

A key finding of eye-tracking research on SLS and subtitling in general is that viewers will engage with the on-screen text automatically (12; 47; 48). Viewers just cannot ignore the subtitles in movies, although they may do so periodically. It is important to note, however, that all these studies were conducted with subjects who could read. Reading along with SLS is preferred because of the efficiency in following and understanding the movie. Evidence of such eye-tracking studies enables us to attribute possible learning outcomes to the subtitles. d’Ydewalle & de Bruycker (12) reported that subtitle reading is inescapable and viewers have little difficulty in distributing visual attention.

Several studies have explored the reading behavior of participants and investigated to what extent they read subtitles (31; 43). These studies primarily limit the eye-tracking metrics to fixation count, fixation time, scan path, and average fixation duration in two areas of interest (AOI): the subtitle band and image. Negi & Mitra (39) eye-tracking study of subtitled videos revealed that fixation duration can be a useful metric to trace the learning process. Other eye-tracking studies investigate the impact of audio on the reading of subtitles (34) and make recommendations for future cognitive research in the field of audiovisual translation (32).

Reading along with SLS is inescapable, among good readers (13). This pioneering study found that American subjects watching an English movie with SLS and Dutch subjects watching a Dutch movie with SLS, spent considerable time in the subtitle area. Reading SLS was inevitable and comparable for both groups, even though the Dutch subjects had much more experience with subtitles on TV. Reading SLS did not depend much on habit formation. The critical question for us is, would struggling readers, especially those from economically disadvantaged backgrounds, also try and engage automatically with SLS? PlanetRead (44) completed an eye-tracking study of government school children in Grades 2-5 in rural Rajasthan, India, by showing them animated stories with and without SLS. Almost all viewers - beginning, struggling, or good readers - automatically engaged with SLS and could not ignore it.

## User Study

We undertook user studies with participants from remote villages in Rajasthan. We approached an NGO named *Dusra Dashak* that has been working in Abu Road, Rajasthan for over 15 years and has an established on-the-ground connect. The head of the NGO advised us on the villages where we could conduct our eye-tracking study. In the selected villages we first got the required permissions with *Dusra Dashak*’s help and implicit support from the village head to conduct our study. We went door-to-door to mainly identify individuals who had completed their schooling but were still weak readers. As a quick filter, we asked villagers to read a simple Grade 2 level paragraph. Those who struggled to read the text were considered to be weak readers, including poor readers, and selected as study participants. Individually, every participant was asked to see video clips of Hindi songs and dialogs, with and without SLS, on a computer monitor. We collected ocular data while participants watched both versions of the video. In particular, we conducted both statistical and visual data analysis, described in the next section, to address the following four questions.

1.
  What proportion of viewers’ attention is divided between two regions (video and SLS) while watching a video with SLS?
2.
  How can we quantify the amount of engagement with the subtitles?
3.
  How can we differentiate between functional weak readers and poor readers?
4.
  Is there any difference in engagement when the SLS are highlighted?

**Table 1:** Media descriptions of audio-visual clips

Content	Movie Name	Song Name	Duration
Dialogue	Baahubali	Not applicable	64 secs
Song	Besharam	Dil ka jo haal hai	67 secs
Song	Abhimaan	Teri bindiya re	63 secs
Song	Dhadkan	Tum dil ki dhadkan	42 secs
Dialogue	Sholay	Not applicable	100 secs
Song	Karz	Kamaal hai	94 secs

## Procedure

The study procedure had the following steps:

a) We gathered background information on the participant such as age, education, gender, and occupation.
b) An Early Grade Reading Assessment (EGRA) tool was used to assess the participant’s reading level more accurately than the earlier filter test (51).
c) The participant was asked to sit in front of the monitor with screen-based eye-tracker attached to the monitor. They were then asked to undertake the task of watching video clips with and without SLS.
d) Each participant was asked to watch twelve short videos ranging from 42 to 100 seconds. The eye-tracking study was conducted with six unique videos with two versions of each video, one with SLS and the other without SLS. Videos are presented in a randomized order to reduce the order effects. The description of media considered for the study is given in Table 1.
e) We investigated the collected data using statistical and visual analysis (see results section).
f) We developed a web-based hardware agnostic software to analyze eye-tracking data visually. The tool is platform independent and does not require any data-preprocessing, making it easier for users.

### Visualization tool

The tool investigates important eye-tracking metrics: fixation, saccade, scan path and regressions. The tool explores temporal data without losing spatial information and retains the direction of saccades while analyzing the saccadic length. The eye gaze movements across AOIs are usually studied statistically, and occasionally AOI transitions are examined visually (6). We introduced an interactive method to study and analyze direct and indirect transitions across AOIs. Direct transitions are eye movements from source AOI to target AOI without other AOIs between them. However, if there are one or more AOIs in the eye movement path from source to target AOIs, it is called indirect transition. Unlike existing web-based visualization tools where AOIs are specified as rectangles (3), the proposed tool allows us to define AOI manually in any convex-shaped polygon with four vertices. The software is developed entirely on JavaScript, and the D3.js library was used for rendering visualizations. It is hosted on a python server and tested with Edge, Chrome, and Mozilla Firefox browsers on a Windows operating system. The tool's features are described below.

**Figure 1:**
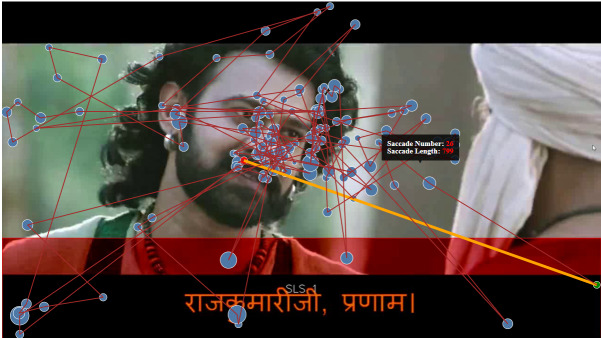
Selection of longest saccade in the main canvas

**Figure 2:**
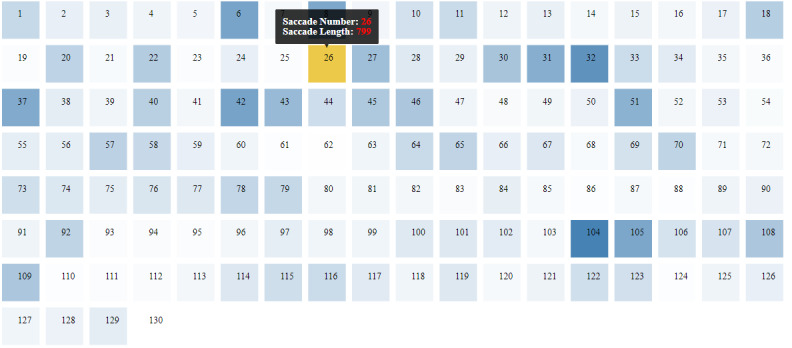
Longest saccade selection with the rectangles

1) AOI visualization: This technique keeps track of the saccadic eye movements between AOIs. Users can manually choose AOIs by defining points of a four-sided polygon from the control panel on the right side of the canvas. After specifying AOIs, we can visualize the number of saccadic movements between AOIs in a bar chart. The tool’s node-link graph shows the frequency of the saccadic movement. Nodes are used for depicting AOIs, and the saccadic movement between them is represented by an edge connecting the nodes. The higher the number of movements among two AOIs, the thicker is the edge connecting the nodes. We can also compare fixations and total durations of AOIs at a particular time interval with a horizontal stacked bar chart used in the tool.

2) Saccade visualization: For investigating saccade amplitudes and direction visually, we developed a two canvas technique in the tool. Visualizing saccades can be a difficult task because of the varying saccadic length. The longest or shortest saccades might be of interest, and the direction of saccades gives an overview of the eye movement in that specific area of the stimulus. It becomes challenging to track both the saccadic length and direction as points get cluttered with gaze plots. We developed a unicolor-coded technique to address this issue. A set of rectangles representing saccades is plotted on a separate graph. Rectangles have identical colors, but their opacity changes according to the length of the corresponding saccade. The more the opacity of the rectangle, the longer is its length. Selecting the rectangle highlights the original saccade in the gaze plot by changing the color and width of the line. The selected saccade's start and target position colors are also changed to red and green, respectively. This technique helps in identifying the direction of the saccade. Both gaze plots and a set of rectangles for saccade visualization are shown in Figures 1 and 2 respectively. It can be noticed that the saccadic movement across the AOIs is one of the biggest saccade amplitude.

3) Fixation and Regression visualization: The tool allows interactive fixation and regression visualization. Regression is a type of eye movement that orients backward with respect to normal reading behavior. The software separates regressions from normal saccades by highlighting them with different colors. Fixations are shown as scatter plots where each circle represents one gaze fixation. The size of the circle depends on the duration of the fixation, bigger circles depict higher durations. An interactive brush is used to select a range of fixations, the size of the brush can be adjusted vertically. The range of the brush corresponds to the timeline of the task. For example, if a user want to explore fixations of a particular time segment of the task, he or she can adjust the brush so that it corresponds to that time segment. The technique would help users to reduce the clutter and concentrate only on specific fixations.

### Participants

We selected overall 136 participants who are weak readers and our goal was to track their eye movements while they are watching Hindi film-based songs and dialog clips, with and without SLS. The sex and reading ability profile is given in Table 1.

**Table 2:** Media descriptions of audio-visual clips

	Female	Male	Total
Weak readers (Total)	81	55	136
Poor readers	38	19	57
Functional weak readers	43	36	79

All the participants were above 14 years of age, except for one. The average age was 24 years. Parents accompanied children below 18 years to the eye-tracking location set up in the community and gave oral permission since most of them were non-literate themselves. The average grade completed was 7.9 with a standard deviation of 3.3. Over 32% in 7^th^ and 27% in 8^th^ grade are not able to read a 2^nd^ grade text in rural India (1).

### Materials

A Gazepoint 3 screen mounted eye tracker with an accuracy of 0.5º-1º of visual angle, sampling rate of 60 Hz, and an average calibration error of 0.5-1 degrees of visual angle was used to collect gaze-based data. A 19-inch computer monitor was used to display the videos for participants. An EGRA (51) was used to assess the participants’ reading ability. The test is in increasing order of difficulty starting with characters and finishing with a simple story. The subsection below explains the test in detail.

**Figure 3:**
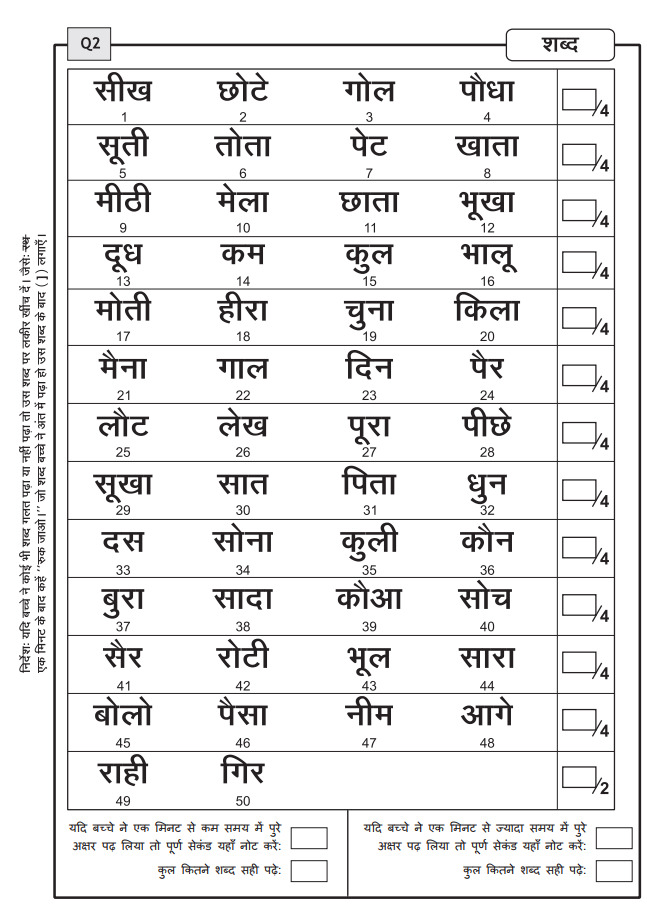
Sample Hindi reading test of 50 words

The reading test, pitched at 2^nd^ Grade level, comprises five simple exercises with increasing difficulty. The participants were asked to read:

- Hindi syllabary in random order comprising 52 syllables.
- 50 meaningful words in Hindi (Figure 3).
- 50 meaningless words in Hindi.
- Four simple Hindi sentences in Hindi with 19 words in total.
- A Hindi story of 65 words.

The participants were given 60 seconds for every exercise. We noted the number of correct letters or words read. The participants who read correctly more than 80% of the words in all the exercises were considered to be functional weak readers, and others were considered poor readers.

### Data collection and analysis

**Figure 4:**
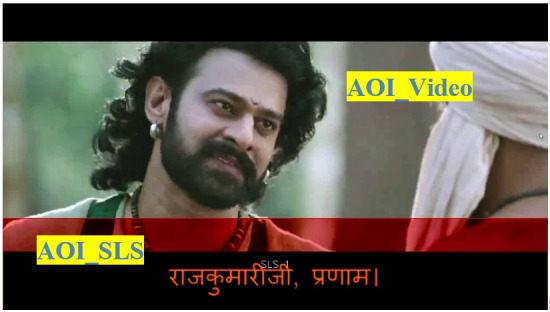
Video and SLS region considered as first AOI set

**Figure 5:**
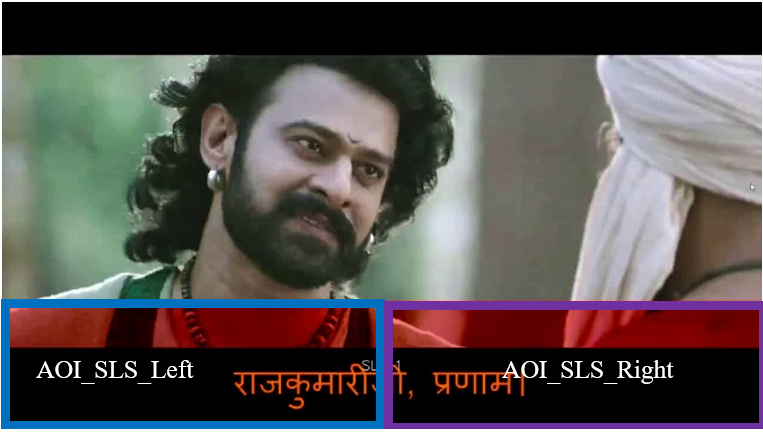
Two parts of the SLS area are second AOI se

All 136 participants were asked to sit in front of a computer monitor at 100 cm from the monitor and 60 cm from a screen-based eye-tracker. They were then asked to calibrate with the eye-tracker with a 9-point calibration routine built into the eye tracking software. It required participants to follow dots on the screen. This ensured accurate eye-tracking across the entire screen for all participants. Participants were then asked to watch the videos and the order of the videos was randomized to reduce any learning impacts. The eye-tracking software collected data while participants were undertaking the task. Eye-tracking data were primarily analyzed with respect to AOIs corresponding to the videos shown to users. We considered two sets of AOIs for the analysis. In the first set, the screen is divided into two AOIs: the first AOI is the region with video (2/3rd of the screen) and the second AOI is the region with SLS, as shown in Figure 4. From the visualization tool, we found that fixations of poor readers tend to be more in the left region than the right region of the subtitle area. Based on this observation, we have split the SLS region into two equal parts (left and right regions) to delve deeper into the pattern, as shown in Figure 5.

## Results

This section lists the indicators and parameters that can answer the questions addressed in section 1 on the resulting reading behavior from SLS. In the subsections below, we first list the parameters and then discuss the results of our statistical analysis to justify why the selected indicators can address our research questions. We also report a visual analysis of eye tracking data to support the results. For the analysis, we calculated the average values of the indicators or parameters for all 136 participants. We prepared a table of 12 columns corresponding to two versions (SLS and No SLS) of six unique videos and 136 columns corresponding to 136 participants.

We further analyzed the data statistically with two independent variables and each variable has two levels: a) Video: with and without SLS, and b) AOIs: AOI_SLS and AOI_Video. In particular we undertook Video(2) × AOI(2) repeated measure ANOVA for identifying statistically significant differences between the experimental conditions. We report the statistical results in the sections below along with other results.

### What proportion of viewers’ attention is divided between two regions (video and SLS) while watching a video with SLS

Indicators considered for this question are explained below:

1) Change in the number of fixations and saccades in AOI_Video

**Figure 6:**
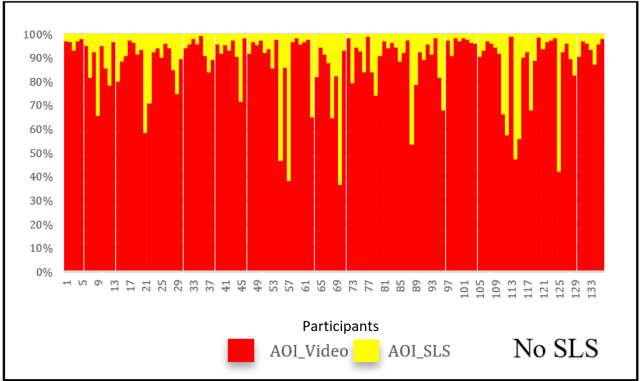
Percentage of fixations in video without SLS

**Figure 7:**
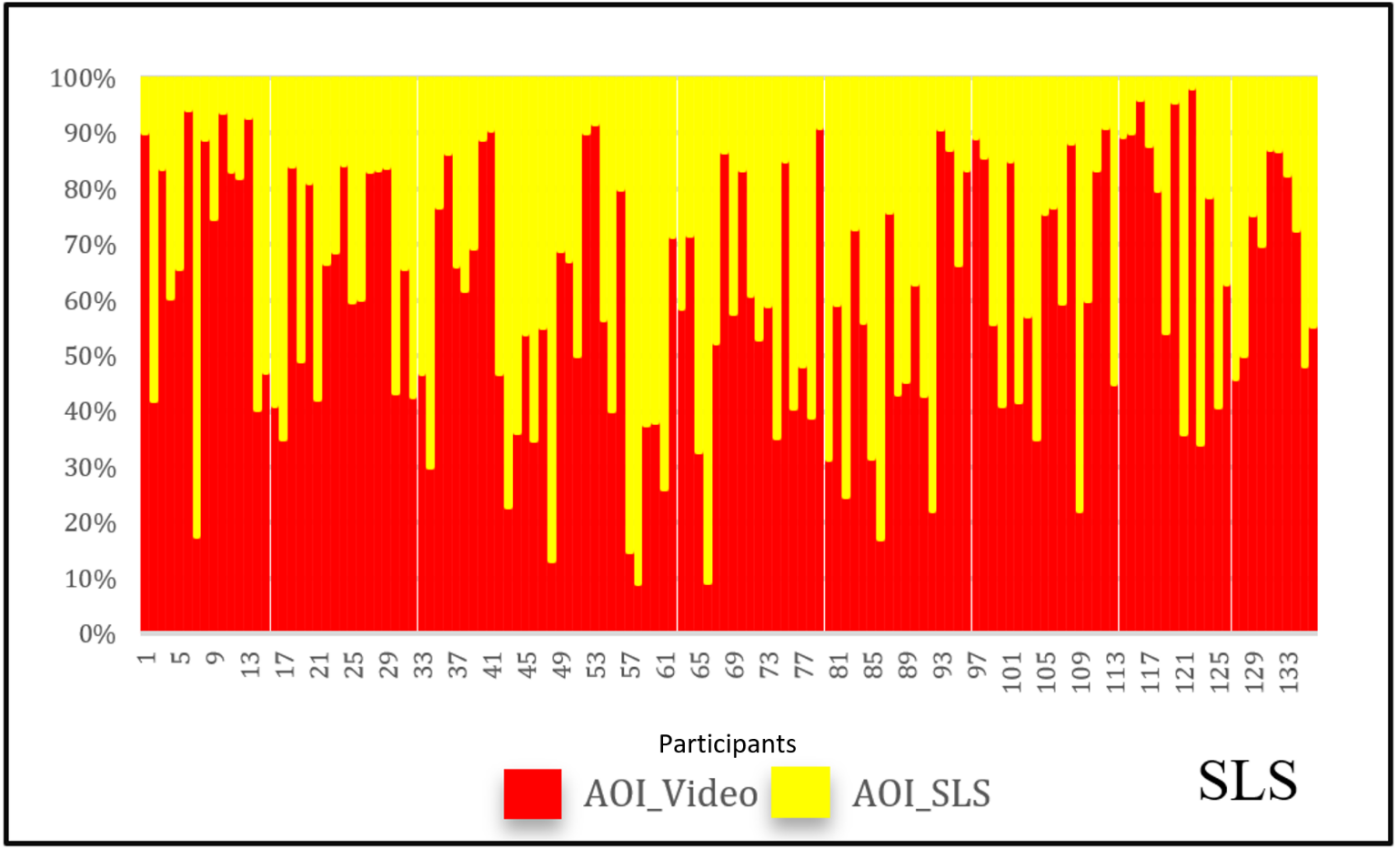
Percentage of fixations in video with SLS

Participants who try to read subtitles while watching a video with SLS are expected to devote more time to AOI_SLS. Participants' fixations and saccades at AOI_Video would be less if they try to scan subtitles on AOI_SLS while watching a video with SLS. A consideration of both fixations and saccades ensures that participants were indeed trying to concentrate at the region rather than just moving their eyes. Figures 6 and 7 show how fixation counts can change in both the AOIs for the same video, without SLS and with SLS. The change of participants’ fixations and saccades at AOI_Video was calculated by subtracting the fixation numbers in the video without SLS from those in the video with SLS. We calculated the change for each pair of videos (with and without SLS) across all the participants and the videos. Table 3 illustrates the average percentage of change in eye movements across all the participants for six pairs. In four out of the six pairs of videos the percentage change in the eye movements is more than 25%. We also undertook paired-samples t-test for each pair of videos to statistically investigate the change of eye movements in AOI_Video. The results listed in Table 4 indicates that the eye movements in AOI_Video are significantly different between each pair of all six videos.

**Table 3:** Changes in eye movements in AOI_Video

Pair No.	Movie name	Percentage change
1	Baahubali	27.87 %
2	Besharam	19.48 %
3	Abhimaan	34.35 %
4	Dhadkan	22.84 %
5	Sholay	27.55 %
6	Karz	25 %

**Table 4:** Paired-samples t-test

Pair No.	Movie name	Result
1	Baahubali	t(132) = -11.36, p<0.05
2	Besharam	t(132) = 7.85, p<0.05
3	Abhimaan	t(132) = 14.3, p<0.05
4	Dhadkan	t(132) = 8.78, p<0.05
5	Sholay	t(132) = -12.52, p<0.05
6	Karz	t(132) = 9.86, p<0.05

2) Total time spent in AOI_Video

The time spent by participants in AOI_Video while watching a video without SLS is expected to be more than a video with SLS. Based on this assumption, the total time spent could be helpful in identifying readers' attention in both the AOIs. We computed the differences in time spent in AOI_Video between the pair of videos (with and without subtitles) as this is the constant AOI in both the situations. The difference would express the divided attention of participant due to the inclusion of subtitles. Table 5 shows the average time differences in AOI_Video across all participants for each pair of video. After undertaking repeated measure ANOVA, we found significant difference for the interaction effects of video × AOI: F(1, 135) = 287.42, $\eta^{2}=0.68,$ p < 0.05. We also found that video AOI are significantly different for the time spent. F(1, 135) = 754.77, $\eta^{2}=0.84,$ p < 0.05. We also undertook a pairwise comparison test like least significant difference (LSD) and found that AOI_Video in the video without subtitle is significantly different from the video with subtitle (p < 0.05).

**Table 5:** Differences in time spent in AOI_Video (without SLS minus with SLS)

Movie name	Total time (secs)	Time differences (secs)
Baahubali	64	15.24
Besharam	67	10.91
Abhimaan	63	17.55
Dhadkan	42	9.07
Sholay	100	23.36
Karz	94	21

3) Saccades across AOIs or revisits at AOIs

Participants whose attention are divided between the two AOIs while watching videos with SLS would repeatedly visit the subtitle area. While we cannot expect much eye movements between the two AOIs in the videos without SLS as there were no subtitles. However, it is intuitive that the introduction of subtitles would increase the eye movements across the AOIs in videos with SLS. The comparisons of saccades across AOIs would allow us to investigate the quantity with which the participants were trying to visit the subtitle region while watch videos with SLS. We compared the average number of saccades across AOIs for all the participants on all SLS videos and its counterpart. The result conveys that the rate of increase in saccadic eye movement is approximately 60% for videos with SLS as compared to videos without SLS. We undertook 2 × 2 repeated measure ANOVA and found significant difference for the interaction effect of video × AOI: F(1, 135) = 286.53, $\eta^{2}=0.68$, p < 0.05. We further found that video and subtitle AOIs are significantly different for saccades across AOIs F(1, 135) = 329.69, $\eta^{2}=0.7$, p < 0.05. After undertaking the LSD test, we found that the video with SLS is significantly different from the video without SLS for AOI_Video (p < 0.05).

### In what ways can we quantify the amount of engagement with SLS?

The indicators used are explained below:

1) Fixation rate and number of fixations

Fixation rate and the number of fixations in AOI_Video could be good indicators for assessing the amount of engagement. We calculated the average number of fixations and average fixation rate at AOI_Video across all participants for all videos with and without SLS. The fixation rate is calculated as:

$$fixation\;rate=\frac{\text{number of fixations}}{total\;durations\;(secs)}$$

**Figure 8:**
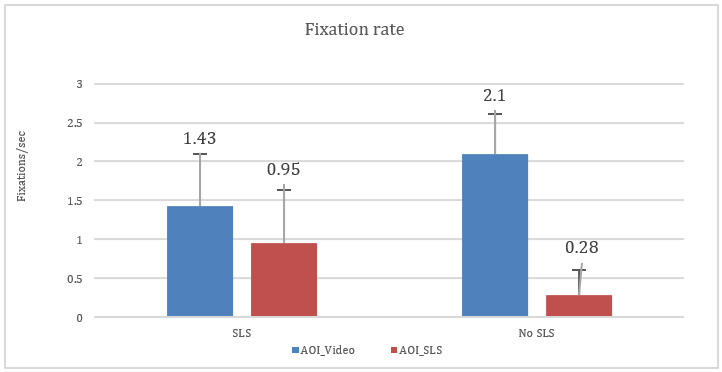
Interaction effect of fixation rate across AOIs. The error bar indicates the standard deviation.

Figure 8 compares the average fixation rate across all participants. We found from repeated measure ANOVA that the interaction effects of video × AOI are significantly different: F(1, 135) = 153.14, $\eta^{2}=0.535,$ p < 0.05. We found that videos with and without SLS are significantly different F(1, 135) = 156.02, $\eta^{2}=0.54,$ p < 0.05.

2) Total durations in AOI_Video

Viewers would be expected to spend more time in AOI_SLS when they try to read the subtitles. Average and total fixation duration could thus be an important variable for quantifying the amount of reader engagement. Higher duration might not always indicate that participants were reading the subtitles. However, it would at least indicate that they were trying to engage in that region while watching the video. We undertook repeated measure ANOVA for total durations in AOI_Video across all participants for all videos and found that there is a significant difference for the interaction effects of video × AOI: F(1, 135) = 287.42, $\eta^{2}=0.68,$ p < 0.05. We also found that video AOI are significantly different for total durations. F(1, 135) = 754.77, $\eta^{2}=0.84,$ p < 0.05.

3) Saccade amplitude at AOI_Video

Eye movements in the video region will be less if participants try to scan through the subtitle while watching the video. It will lead to smaller number of saccades and hence shorter total saccade lengths are expected in the video area and higher total saccade lengths are expected in the SLS area. We investigated the saccade amplitudes for both versions of video and found that saccade amplitude decreases significantly in AOI_Video. Table 6 compares the change of saccade length in AOI_Video for each pair of six videos. We found that the interaction effect of video × AOI are significantly different: F(1, 135) = 221.99, $\eta^{2}=0.62,\;$p < 0.05. The videos with and without SLS are significantly different for saccade length F(1, 135) = 16.82, $\eta^{2}=0.11,$ p < 0.05. After undertaking LSD test, we found that both versions of video are significantly different with respect to AOI Video (p < 0.05).

**Table 6:** Changes in saccade amplitude (pixels) in AOI_Video

Pair No.	Movie name	Percentage of change
1	Baahubali	26.82 %
2	Besharam	19.22 %
3	Abhimaan	31.48 %
4	Dhadkan	19.95 %
5	Sholay	25.89 %
6	Karz	23.8 %

### How can we differentiate between functional weak readers and poor readers?

1) Number of regressions at AOI_SLS

Regression is a type of saccadic eye movement that orients backward for normal reading behavior on the screen while reading (7). It is also termed backward eye movement. The standard reading behavior of participants in the Hindi language is from left to right on the screen, and we expect their eye movement in a similar direction. Regression could be an indicator for differentiating between functional weak readers (FWR) and poor readers (PR). We expect poor readers to have higher number of regressions than the functional weak readers, as poor readers would read with greater difficulty than their counterparts. We compared the average number of regressions for FWR and PR only on videos with SLS and observed that number of regressions for both the groups are almost similar. The results of the comparison are listed in Table 7. As regressions in AOI_Video would not help us in identifying the reading behaviour of participants, we analysed regressions only at AOI_SLS. We also undertook paired sample t-test and did not find any significant difference between the two groups.

**Table 7:** Average number of regressions

Movie name	PR	FWR
Baahubali	13.96	16.67
Besharam	11.98	12.36
Abhimaan	17.14	17.92
Dhadkan	7.89	8.24
Sholay	23.59	26.97
Karz	19.56	19.15

2) Fixations at left AOI_SLS and right AOI_SLS

**Figure 9:**
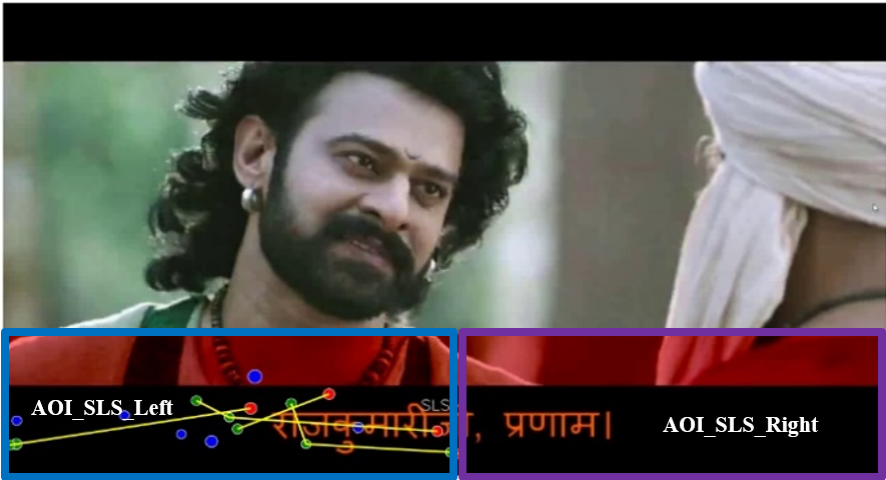
Fixations at AOI_SLS (left/right) of a poor reader

**Figure 10:**
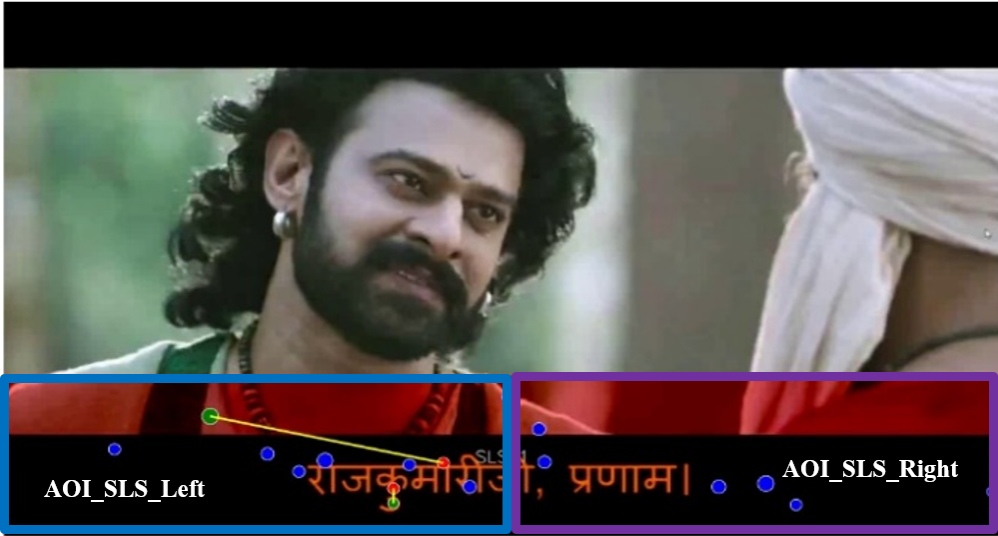
Fixations at AOI_SLS (left/right) of a functional reader

We noticed from the visualization tool, as shown in Figures 9 and 10, that poor readers have relatively more fixations at the left part of the subtitle area than functionally weak readers. We found that the average fixation rate at AOI_SLS_Left is relatively more for poor readers than functional weak readers in all videos. However, the difference is not significantly high as can be seen from Table 8. We also undertook paired samples t-test and did not find any significant difference between the two groups.

**Table 8:** Comparisons of fixations at left AOI_SLS_Left

Movie name	PR	FWR
Baahubali	61 %	52.8 %
Besharam	60 %	53 %
Abhimaan	50.2 %	40.5 %
Dhadkan	56 %	50 %
Sholay	57 %	54 %
Karz	57 %	54 %

### Is there any difference in engagement when the SLS are highlighted?

Among the six videos with SLS, the subtitle words of two videos are not highlighted and the words of four videos are highlighted. We considered the number of fixations and durations for analysis.

1) Number of fixations and durations

As stated earlier, the number of fixations and durations could be a good indicator for identifying reading engagement. We used these same variables for comparing our two design versions of SLS. We did not find any considerable difference among the subtitle designs for both the parameters.

## Discussion

Our study is unique in several respects. First, a number of eye-tracking studies have established automatic reading behavior while watching video content, but, in almost all these studies, the subjects were good readers. Our subjects were all weak and many of them poor readers and we established that a majority of them also try to read along with SLS on video.

Second, eye-tracking studies in India generally, and of video consumption in particular, are rare. With a billion TV viewers and 356 million mobile video viewers in 2021 (21), and growing fast, eye gaze research on how viewers consume video content, with or without subtitles, or interact with screens, is a fertile area for study.

Third, to our knowledge, there is no eye gaze study conducted with subjects from rural India. Perhaps the reason is that it may seem difficult to transport and operate eye-tracking equipment in rural areas or bring rural subjects to urban centers. Our study has demonstrated that the former is indeed possible.

Impact measurement studies have found that regular SLS exposure among weak readers leads to reading skill improvement, over time (29; 27). Future studies could explore whether eye gaze measurement can also capture reading skill improvement resulting from SLS exposure over time. How would a subject’s reading of SLS on the same video be different at the baseline and end line, assuming that the subject’s reading skills have improved? The visualization tool proposed in the paper was useful to support the results of the statistical analysis.

This paper presents a detailed eye-tracking study on processing SLS while watching Hindi film songs and dialogs. We analyzed five gaze-based metrics to investigate eye movements of users in the subtitle region of video clips. We also observed the proportion of viewers who try to read along while watching videos. We noticed that regression between poor and functional weak readers are almost similar. We also found significant differences for the interaction effect between the experimental conditions. Weak readers tend to linger more in the left half of the subtitle area. From a policy perspective, a key finding is that both poor readers and functional weak readers tend to engage their eye movements in the subtitle regions with the introduction of SLS in the video clips. We plan to undertake a similar eye-tracking study of SLS film videos to understand changes in eye-gaze patterns when an individual becomes a better reader. This could even be conducted with a subset of our study participants who have measurably improved their reading skills since this study was conducted. Our results could potentially be used to design an adaptive user interface for learning while watching videos with SLS.

### Conclusion

In 2019, SLS became a part of India’s Accessibility Standards (20), mandating that half the entertainment content on TV, in every language, state, and channel, is required to carry SLS by 2025. Our finding that weak readers do try to read along with SLS on films, supports the leveraging of the Accessibility Standards, for both, media access among the DHH and improving the reading skills of over half a billion weak readers in India, an overwhelming majority being female. The Accessibility Standards, if implemented with the intent to benefit all, will then be designed to benefit all. But if they are framed as good only for the hearing impaired, then the implementation risks not leveraging their full potential for the hearing. This perspective is important to keep in mind for policy makers in countries, like India, that are in the process of adopting SLS

Countries that do have captioning on TV might consider re-designing captions with universal visual appeal. By not doing so, they lost out on a massive opportunity to contribute, at scale, to their populations’ reading literacy and language learning. The hearing also want to turn TV captions on for different reasons, as they now do on streaming platforms. Networks might consider making captions available on content in all languages and turning them on by default, especially on children’s programming, like Turn On The Subtitles (TOTS) is campaigning for in the UK. Finally, parents the world over need to know that SLS or captions can make a massive contribution to their child’s reading literacy and language skills. All they have to do is turn them on whenever possible.

### Ethics and Conflict of Interest

The author(s) declare(s) that the article's contents are in agreement with the ethics described in http://biblio.unibe.ch/portale/elibrary/BOP/jemr/ethics.html and that there is no conflict of interest regarding the publication of this paper.
